# Arrhythmogenic Right Ventricular Cardiomyopathy Presenting as Monomorphic Ventricular Tachycardia

**DOI:** 10.1016/j.jaccas.2025.104941

**Published:** 2025-08-27

**Authors:** Fahad Hussain, Andrew Cyr, Vlad Shknevskiy Shusterman, Amit Blumfield, Priyen Patel, Shuojohn Li, Syed Ahmad, Jonathan Willner, Bani Azari, Samit Shah

**Affiliations:** a Department of Internal Medicine, Northwell Health, Manhasset, New York, USA; b Department of Cardiology, Northwell Health, Manhasset, New York, USA

**Keywords:** arrhythmogenic right ventricular cardiomyopathy, cardiomyopathy, electrophysiology, heart failure, ventricular tachycardia

## Abstract

**Introduction:**

Arrhythmogenic right ventricular cardiomyopathy (ARVC) is an autosomal dominant genetic cardiomyopathy characterized by the replacement of right ventricular myocardium with fibrous and adipose tissue, leading to arrhythmias, heart failure, and an increased risk of sudden cardiac death.

**Case Summary:**

A 25-year-old woman without any medical history presented with palpitations after exercise and was found to be in sustained monomorphic ventricular tachycardia. Imaging and presentation met the 2010 modified Task Force Criteria for a diagnosis of ARVC.

**Discussion:**

ARVC can be a devastating cause of heart failure and sudden cardiac death in young adults. Once the diagnosis is confirmed, treatment including exercise restriction, antiarrhythmics, goal-directed medical therapy, ablation, and implantable cardioverter-defibrillator placement can lead to marked improvement in prognosis.

**Take-Home Message:**

Clinicians should consider ARVC in young patients presenting with ventricular tachyarrhythmias and use the 2010 modified Task Force Criteria to guide initial diagnosis before confirming with genetic testing.

## Graphical Abstract

**Figure:**
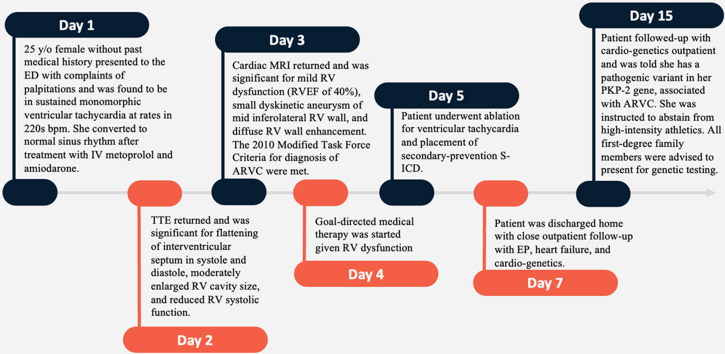

**Visual Summary:**
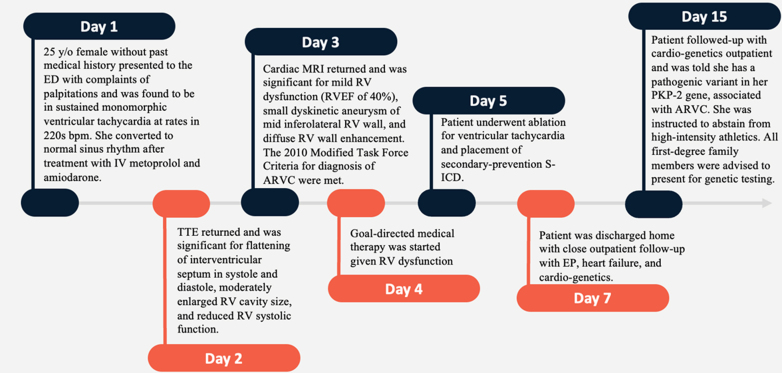
Timeline From Presentation to Conclusion ARVC = arrhythmogenic right ventricular cardiomyopathy; ECG = electrocardiogram; ED = emergency department; EP = electrophysiology; IV = intravenous; MRI = magnetic resonance imaging; RV = right ventricular; RVEF = right ventricular ejection fraction; S-ICD = subcutaneous implantable cardioverter-defibrillator; TTE = transthoracic echocardiogram.

A 25-year-old woman without any past medical history presented to the emergency department with persistent palpitations. After a cycling workout, she experienced the palpitations, and her smart watch showed a persistently elevated heart rate—as high as 115 beats/min—even after hours of rest. She denied a preworkout regimen, use of caffeine, or other supplements. Upon initial evaluation, she was found to have a heart rate in the 220s beats/min, with 12-lead electrocardiogram (ECG) revealing a wide-complex tachycardia consistent with sustained monomorphic ventricular tachycardia and vectors suggestive of right ventricular (RV) outflow tract origin (Figure 1). Valsalva maneuvers were attempted, followed by 6 mg and then 12 mg of adenosine without resolution, but she successfully converted to normal sinus rhythm with 5 mg of intravenous metoprolol and 150 mg of intravenous amiodarone. Her ECG after conversion showed normal sinus rhythm, with T-wave inversions in multiple leads (Figure 2). Her laboratory tests were remarkable for leukocytosis (white blood cell count: 23.6 × 10^9^/ L), elevated high-sensitivity troponin T of 395 ng/L (normal: <51 ng/L), and elevated NT-proBNP of 2,181 pg/mL. Her electrolytes and thyroid stimulating hormone were within normal limits.

**Figure 1:**
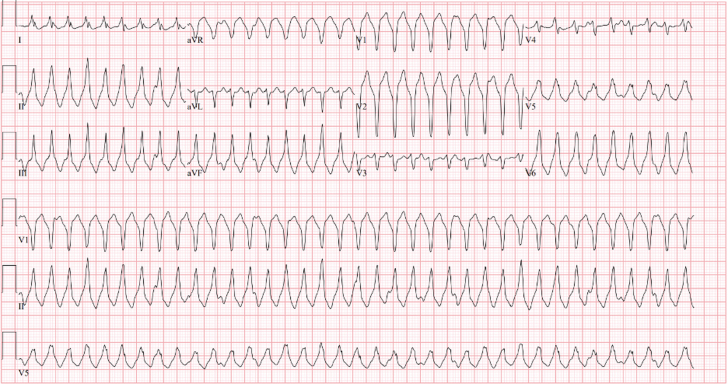
Initial Electrocardiogram on Presentation Electrocardiogram shows a wide complex tachycardia consistent with sustained monomorphic ventricular tachycardia.

**Figure 2:**
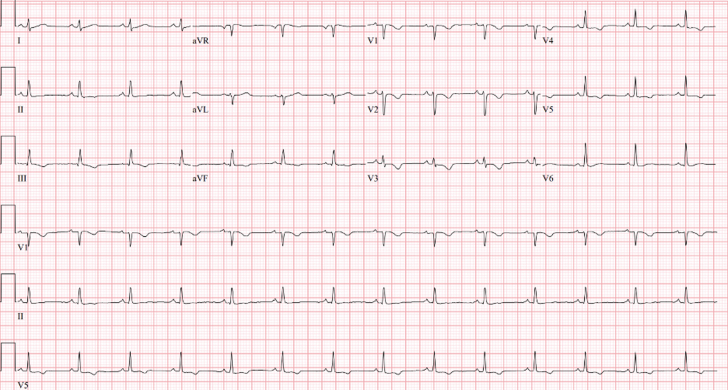
Electrocardiogram After Conversion From Ventricular Tachycardia Electrocardiogram depicts normal sinus rhythm with T-wave inversions in multiple leads.

The patient's social history was notable for an active lifestyle, with participation in cycling classes 3 times a week, scheduled runs twice a week, and involvement in a soccer league. She also competed in 5-k races every 3 to 4 months and previously ran a half-marathon. Her family history was significant for Ashkenazi Jewish descent and sudden death in a paternal grandfather from presumed myocardial infarction at age 60.

She was monitored on telemetry without further episodes of arrhythmia. Transthoracic echocardiogram showed a left ventricular ejection fraction of 65%, mildly increased left ventricular wall thickness, flattening of the interventricular septum in systole and diastole, moderate RV dilatation, and moderate RV dysfunction, as demonstrated by a tricuspid annular plane systolic excursion of 1.3 cm. Cardiac magnetic resonance imaging (MRI) showed the RV size to be within upper normal limits (RV end-diastolic volume index: 100 mL/m^2^; normal range: 57-108 mL/m^2^), mild RV dysfunction (RV ejection fraction: 40%), small dyskinetic aneurysm of the mid inferolateral RV wall, diffuse RV wall enhancement, subepicardial enhancement of the mid anterolateral, lateral, and inferolateral myocardium in a nonischemic distribution, and flattening of interventricular septum with D-shaped left ventricle, consistent with RV overload (Figure 3, Figure 4, Figure 5).

**Figure 3:**
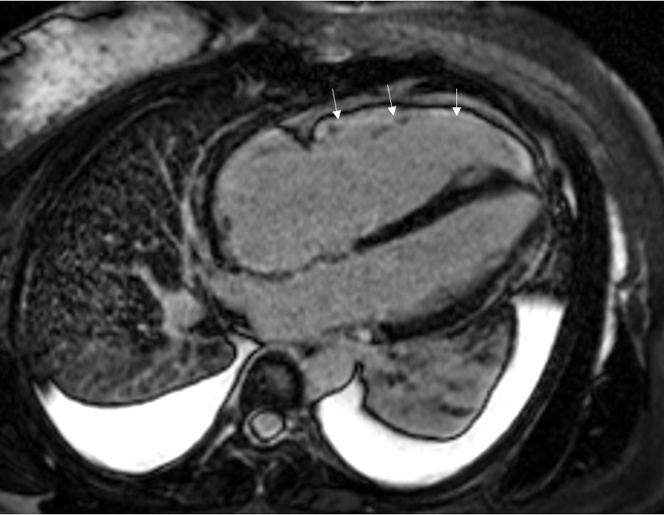
4-Chamber Delayed Enhancement Cardiac MRI Image demonstrating diffuse late gadolinium enhancement of the lateral right ventricular wall. Note right atrial and right ventricular enlargement and small pleural effusions. The arrows indicate the areas of late gadolinium enhancement. MRI = magnetic resonance imaging.

**Figure 4:**
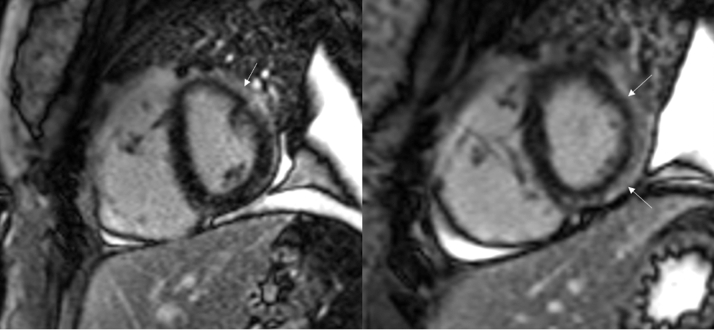
Short-Axis Delayed Enhancement Cardiac MRI Through the Midcavity The images demonstrate diffuse subepicardial late gadolinium enhancement of the anterolateral and inferolateral left ventricular myocardium as well as the adjacent pericardium. Note the D-shaped configuration of the left ventricle with interventricular septal flattening in the setting of right ventricular overload. The arrows indicated the areas of late gadolinium enhancement. MRI = magnetic resonance imaging.

**Figure 5:**
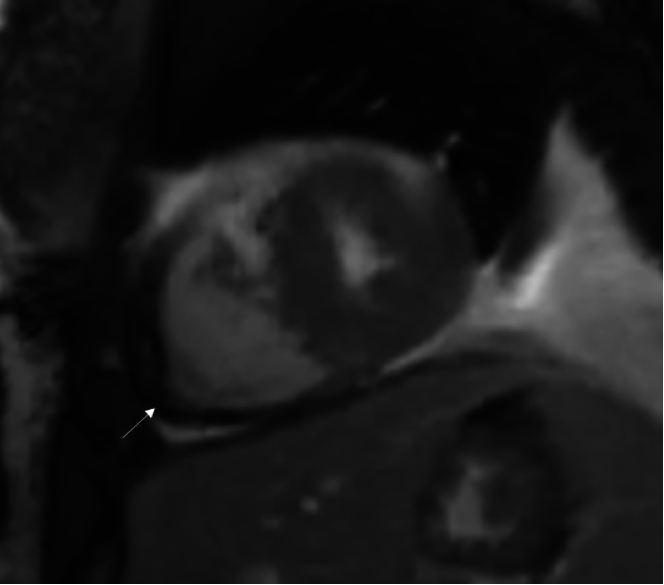
TrueFISP Cine Cardiac MRI in Short-Axis View There is a small aneurysm of the mid inferolateral right ventricular wall that demonstrates dyskinetic motion on cine imaging. The arrow indicates the aneurysmal area. MRI = magnetic resonance imaging.

Given these findings, the patient met the 2010 modified Task Force Criteria for diagnosis of ARVC.^1^ The decision was made to pursue ventricular tachycardia ablation, with placement of an implantable cardioverter-defibrillator (ICD) for secondary prevention. Both procedures were performed during the initial hospitalization, without complication. Before hospital discharge, she was initiated on goal-directed medical therapy with metoprolol succinate and spironolactone for RV dysfunction as well as suggestion of potential left ventricular involvement as described by the MRI. Genetic testing was sent before discharge and resulted positive for *PKP2* gene variant, which is highly associated with ARVC. She was instructed to abstain from high-intensity athletics and to consider in vitro fertilization with preimplantation genetic testing of embryos for future pregnancies. First-degree family members were scheduled for genetic testing.

## Discussion

This case highlights a young, athletic woman who presented with palpitations, was found to have monomorphic ventricular tachycardia, and ultimately met the criteria for ARVC after a series of diagnostic tests, including electrocardiography, echocardiography, and cardiac MRI. The diagnosis was confirmed according to the 2010 modified Task Force Criteria (Table 1) and was further corroborated with positive genetic testing.

**Table 1:** The 2010 Modified Task Force Criteria for Diagnosis of ARVC

Category	Major Criteria	Minor Criteria
I. Global/Regional dysfunction and structural alterations	By 2D echocardiogram: regional RV akinesis, dyskinesia, or aneurysm as well as one of the following: • PLAX RV outflow tract: ≥32 mm (PLAX/BSA: ≥19 mm/m^2^) • PSAX RV outflow tract: ≥36 mm (PSAX/BSA: ≥21 mm/m^2^) • Fractional area change: ≤33% By MRI: regional RV akinesis, dyskinesia, or dyssynchronous contraction as well as one of the following: • RVEDV/BSA: ≥110 mL/m^2^ (male) or≥100 mL/m^2^ (female) • RV ejection fraction: ≤40% By RV angiography: regional RV akinesis, dyskinesia, or aneurysm	By 2D echocardiogram: regional RV akinesis or dyskinesia as well as one of the following: • PLAX RV outflow tract: 29-32 mm (PLAX/BSA: 16-19 mm/m^2^) • PSAX RV outflow tract: 32-36 mm (PSAX/BSA: 18-21 mm/m^2^) • Fractional area change: 33%-40% By MRI: regional RV akinesis, dyskinesia, or dyssynchronous contraction as well as one of the following: • RVEDV/BSA: 100-110 mL/m^2^ (male) or 90-100 mL/m^2^ (female) • RV ejection fraction: 40%- 45%
II. Tissue characterization	Residual myocytes <60% (or <50% if estimated) with fibrous replacement of RV free wall (with or without fatty infiltration) on endomyocardial biopsy	Residual myocytes 60% to 75% (or 50%-60% if estimated) with fibrous replacement of RV free wall (with or without fatty infiltration) on endomyocardial biopsy
III. Repolarization abnormalities	T-wave inversions in V_1_ to V_3_ (or beyond), age >14 y, in the absence of complete RBBB	T-wave inversions in: • V_1_ and V_2_ (age >14 y, no complete RBBB) • V_4_-V_6_ (no RBBB) • V_1_-V_4_ (if complete RBBB is present)
IV. Depolarization/Conduction abnormalities	Epsilon wave (reproducible low-amplitude signals at end of QRS) in V_1_ to V_3_	Late potentials on signal-averaged ECG in ≥1 of 3 parameters (in the absence of QRS ≥110 ms): • Filtered QRS duration ≥114 ms • Low-amplitude signal duration ≥38 ms • Root mean square voltage of last 40 ms ≤20 μV OR: terminal activation duration of QRS ≥55 ms (from nadir of S to QRS end) in V1-V3 (if no RBBB)
V. Arrhythmias	VT (sustained or nonsustained) of LBBB morphology with superior axis (negative or indeterminate QRS in II, III, aVF; positive in aVL)	VT (sustained or nonsustained) with: • RV outflow tract configuration (LBBB morphology, inferior axis) • Unknown axis • >500 PVCs per 24 h (Holter ECG)
VI. Family history	• Confirmed ARVC in a first-degree relative who meets the Task Force Criteria • ARVC confirmed pathologically at autopsy or surgery in a first-degree relative • Pathogenic mutation associated with ARVC in the patient under evaluation	• History of ARVC in a first-degree relative, but Task Force Criteria not confirmed • Premature sudden cardiac death (<35 y) suspected due to ARVC in a first-degree relative • ARVC confirmed pathologically or by Task Force Criteria in a second-degree relative

From Wang et al.^1^

2D = 2-dimensional; ARVC = arrhythmogenic right ventricular cardiomyopathy; BSA = body surface area; ECG = electrocardiogram; LBBB = left bundle branch block; PLAX = parasternal long-axis view; PSAX = parasternal short-axis view; PVC = premature ventricular contraction; RBBB = right bundle branch block; RV = right ventricular; RVEDV = right ventricular end-diastolic volume; VT = ventricular tachycardia.

Although ARVC can be diagnosed at any age, it predominantly presents in young patients, particularly athletes. Exercise-induced arrhythmias are common in ARVC, and several studies have demonstrated that high-intensity exercise increases adipose deposition in the myocardium and is associated with an increased risk of arrhythmic events.^2^, ^3^, ^4^ Common presenting symptoms include palpitations, lightheadedness, and syncope with exercise; however, many patients do not experience symptoms, and about 20% of patients diagnosed with ARVC first present with cardiac arrest.^5^

ARVC is more commonly a disease of the right ventricle, but biventricular or left ventricular involvement is often seen.^5^ As a result, the naming convention is shifting toward “arrhythmogenic cardiomyopathy.” It is predominantly caused by mutations in desmosome-specific genes. Desmosomes maintain cell-cell integrity between cardiac myocytes, and when dysfunctional, they can lead to inflammation, fibrosis, cell death, and ultimately replacement of healthy myocardium with fibrofatty tissue.^6^ Currently, the genes known to be associated with ARVC are *PKP2*, *DSP*, *DSG2*, *DSC2*, *JUP*, *TMEM43*, *DES*, and *PLN*.^5^ The patient in this case was found to have the PKP2 c.2146-1G>C (splice acceptor) pathogenic gene variant, which alters splicing in codon 10 and results in production of a nonfunctional protein or early stop codon. *PKP2* variants are the most common, accounting for 20% to 46% of ARVC cases. *DSP* and *DSG2* are the next most common variants, each accounting for 10% of cases.^5^ ARVC shows an autosomal dominant inheritance pattern, with variable penetrance and incomplete expression.^5^ If a parent carries the pathogenic variant, their offspring will have a 50% chance of inheriting it. However, given variable penetrance, it is possible that some individuals with the mutation may never develop ARVC. Additionally, patients with the mutation may vary in the degree to which they express the symptoms, even within the same family. For these reasons, all first-degree relatives of a patient diagnosed with ARVC should undergo genetic testing.

**Figure 6:**
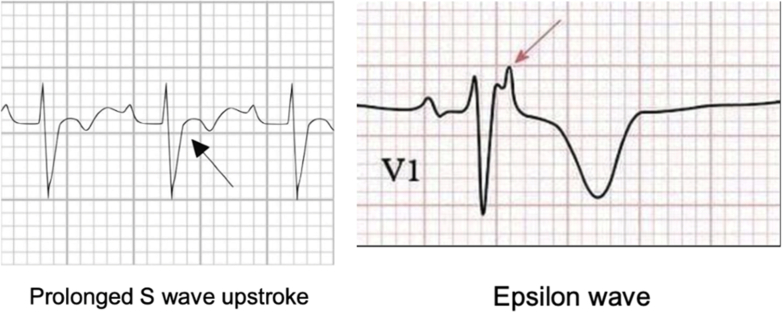
Common ECG Findings of ARVC Findings included prolonged S-wave upstroke (55 ms in V_1_ to V_3_; from S-wave to baseline) and epsilon wave (small positive deflection at the end of the QRS complex in leads V_1_ to V_3_).

The diagnosis of ARVC is based on the 2010 modified Task Force Criteria (Table 1).^1^ For brevity, our discussion will address the criteria more generally. A patient must portray at least 2 major criteria, 1 major and 2 minor criteria, or 4 minor criteria from different categories, including clinical, ECG, imaging, histopathologic, and family history.^1^ Clinical criteria include ventricular arrhythmias and family history of ARVC or sudden cardiac death. ECG criteria include the presence of epsilon waves (Figure 6), frequent ventricular ectopy, and ventricular arrhythmias. Imaging criteria include RV dilatation, hypokinesia, aneurysms, or RV dysfunction. Histopathologic criteria are based on the identification of fibrofatty replacement in the myocardium, and family history criteria include ARVC or sudden cardiac death in a first-degree relative.^1^ Our patient was found to be meeting at least 2 major criteria in the clinical (ventricular arrhythmia) and imaging (RV wall aneurysm) sections. Genetic testing confirmed our suspicion for ARVC.

Management of ARVC is focused on controlling arrhythmias, preventing sudden cardiac death, and addressing heart failure. Given that exercise can provoke arrhythmias and accelerate disease progression in ARVC, patients are asked to restrict exercise to about 650 metabolic equivalents per year. This correlates to no more than 30 minutes of brisk walking daily.^5^ Restriction of exercise in young, otherwise healthy patients can often be a difficult adjustment to make. Sotalol is typically the first-line choice for pharmacological suppression of arrhythmias, and catheter ablation can be considered for patients who have recurrent arrhythmias despite appropriate antiarrhythmic therapy or to reduce the likelihood of device therapy.^7^ There is a high rate of ventricular tachycardia recurrence despite ablation. ICDs can be implanted and are the most effective preventive strategy for sudden cardiac death. Subcutaneous ICDs are frequently used given the relatively young population presenting with ARVC. Indications for ICD placement in ARVC include history of cardiac arrest secondary to ventricular arrhythmia, symptomatic ventricular tachycardia that cannot be controlled with antiarrhythmic medications, severe dysfunction of the right or left ventricle and poor tolerance to ventricular tachycardia, episodes of nonsustained ventricular tachycardia and syncope that are suspected to be of arrhythmic origin, and sudden cardiac death of a first-degree relative.^5^ The other component to management of these patients is goal-directed medical therapy for heart failure. All patients with ARVC should be started on a beta-blocker to reduce RV wall strain and also help suppress arrhythmias.^5^ Patients should also be introduced to further guideline-directed medical therapy (angiotensin-converting enzyme inhibitors/angiotensin receptor blockers/angiotensin receptor-neprilysin inhibitors, mineralocorticoid receptor antagonists, and SGLT-2 inhibitors) as tolerated if associated with left ventricular dysfunction. In the case of profound ventricular failure unable to be managed pharmacologically or ventricular arrhythmias refractory to treatment, heart transplant is considered as the final option. The 10-year posttransplant survival rate for patients with ARVC is about 77%.^5^ With appropriate management and follow-up however, most patients are able to avoid heart transplantation; long-term outcomes of index patients suggest that only 4% of patients will require heart transplant, and 89% can be expected to be alive after a median of 7 years, with the vast majority requiring an ICD for prophylaxis.^8^ The next frontier in the treatment of ARVC is likely to be gene therapy. Preclinical studies using adeno-associated virus vector–mediated delivery of the human *PKP2* gene to *PKP2*-deficient mice prevented RV dilation, left ventricular functional decline, and reduced arrhythmia burden.^9^ This therapy is currently undergoing phase 1/2 study in humans.^10^

## Conclusions

ARVC should be considered in the differential diagnosis for any young patient presenting with ventricular tachyarrhythmias and structural cardiac abnormalities, particularly if there is a family history or genetic predisposition. Early diagnosis and intervention, including ICD implantation and suppression of arrhythmias, are essential in improving long-term outcomes and preventing sudden cardiac death in these patients.

## Funding Support and Author Disclosures

The authors have reported that they have no relationships relevant to the contents of this paper to disclose.

**Take-Home Message**

• Clinicians should consider ARVC in young patients presenting with ventricular tachyarrhythmias and utilize the 2010 modified Task Force Criteria to guide initial diagnosis before confirming with genetic testing.
